# LDLR-Mediated Targeting and Productive Uptake of siRNA-Peptide Ligand Conjugates In Vitro and In Vivo

**DOI:** 10.3390/pharmaceutics16040548

**Published:** 2024-04-17

**Authors:** Baptiste Broc, Karine Varini, Rose Sonnette, Belinda Pecqueux, Florian Benoist, Maxime Masse, Yasmine Mechioukhi, Géraldine Ferracci, Jamal Temsamani, Michel Khrestchatisky, Guillaume Jacquot, Pascaline Lécorché

**Affiliations:** 1 Vect-Horus S.A.S, Faculté des Sciences Médicales et Paramédicales Secteur Timone, 13385 Marseille, France; 2 Aix-Marseille Univ, CNRS, INP, Inst Neurophysiopathol, 13005 Marseille, France

**Keywords:** peptide, siRNA, siRNA-peptide conjugate, LDLR, knock-down, in vitro, in vivo

## Abstract

Small RNA molecules such as microRNA and small interfering RNA (siRNA) have become promising therapeutic agents because of their specificity and their potential to modulate gene expression. Any gene of interest can be potentially up- or down-regulated, making RNA-based technology the healthcare breakthrough of our era. However, the functional and specific delivery of siRNAs into tissues of interest and into the cytosol of target cells remains highly challenging, mainly due to the lack of efficient and selective delivery systems. Among the variety of carriers for siRNA delivery, peptides have become essential candidates because of their high selectivity, stability, and conjugation versatility. Here, we describe the development of molecules encompassing siRNAs against *SOD1*, conjugated to peptides that target the low-density lipoprotein receptor (LDLR), and their biological evaluation both in vitro and in vivo.

## 1. Introduction

RNA interference (RNAi) has become a robust tool to silence, in a highly specific manner, genes of interest in mammalian cells. The underlying mechanism of RNAi is based on the uptake by the cytoplasmic RNA-induced silencing complex (RISC) of small (21–24 nucleotides in length) interfering RNA (siRNA) that bind in a specific base-pairing manner with their complementary mRNA sequence, thereby triggering their degradation and suppressing expression of disease-causing proteins [1,2,3]. Theoretically, therapeutic siRNAs have the potential to treat any gene-related pathology thanks to their potency [4], specificity [5], and duration of effect [6]. 

However, because of their small size within the limit of active filtration and highly hydrophilic nature, naked-stabilized siRNAs spontaneously accumulate in proximal epithelia of kidneys when administered systemically and are rapidly and extensively cleared (>90% within 2–5 min), essentially through the kidney [7,8]. The specific delivery of siRNAs in tissues of interest remains a scientific lock, mainly due to the lack of efficient and selective delivery systems [9,10]. From lipid and polymer nanoparticles [11,12,13,14] that encapsulate free siRNA to well-defined and stable molecular Ligand-siRNA conjugates [15,16], a wide variety of delivery systems are currently on the study bench to fully unlock the potential of siRNAs.

While numerous cell surface markers can be targeted to allow tissue-specific enrichment of carrier-drug conjugates [17,18,19], only a few examples of ligand/receptor pairs were shown to support efficient and tissue-selective functional delivery of siRNAs [20]. Following the clinical success of N-acetylgalactosamine (GalNAc)-siRNA conjugates, which efficiently and specifically target liver hepatocyte asialoglycoprotein receptors (ASGPR)s allowing potent functional uptake and gene knock-down (KD) [21,22], other receptors with constitutive and/or ligand-mediated endocytosis represent attractive cell-surface receptors for siRNA delivery into intracellular compartments [23]. Among them, the LDL receptor (LDLR) is an attractive cell surface target. It is endowed with high recycling activity leading to high uptake potential of circulating ligands, as evidenced by its fundamental role in LDL-cholesterol plasma clearance [24]. The underlying subcellular mechanisms have been well described and involve efficient endosomal release of LDL in the mildly acidic environment (pH 6.0) of early sorting endosomes (SE) [25,26,27]. Because the expression levels of LDLR and its uptake capacity correlate with the need for LDL-derived cholesterol in major biological processes, the LDLR displays differential expression among organs that may be exploited in pathophysiological conditions, including cancer. Given the potential of LDLR as a relevant cell surface receptor in targeted drug delivery approaches, we identified and optimized a family of peptide-based vectors that target the LDLR and that meet the following requirements: (i) high selectivity and nanomolar affinity for both the rodent and human LDLR to allow preclinical studies, while managing risk for further clinical studies; (ii) minimal sized 8 amino acid cyclic peptides, chemically optimized for increased stability; (iii) absence of competition with LDL, a major endogenous ligand of the LDLR; (iv) proven conjugation versatility while retaining LDLR uptake capacity, allowing delivery of a variety of cargos ranging from small organic molecules, peptides, siRNAs, and proteins, including antibodies; and (v) in vivo validation of their specific LDLR-dependent tissue distribution in wild-type or ldlr−/− mice [28,29].

Among the family of peptide-based vectors we identified, the VH4127 (cyclo[(D)-Cys-Met-Thz-Arg-Leu-Arg-Gly-Pen]) peptide ligand used in this study was described elsewhere [28]. Briefly, it was obtained by high-throughput screening of phage display peptide libraries on engineered cell lines expressing the mouse and human LDLR, and was further chemically optimized for optimal LDLR-binding affinity and plasma stability [30]. The VH4127 peptide contains three non-natural amino-acids D-Cys, Thz, and Pen (at positions 1, 3, and 8, respectively), and a disulfide bridge between D-Cys and Pen side chains. LDLR-binding kinetics (on-rate, k_on_, and off-rate, k_off_) of peptide VH4127 assessed using surface plasmon resonance (SPR) allowed determination of its equilibrium dissociation constant (Kd = 40.1 nM). In vitro plasma stability of the VH4127 peptide was evaluated using incubation at 2 mM in freshly collected mouse blood at 37 °C. LC-MS/MS analysis performed on the plasma fractions at the end of indicated time points led to an estimated half-life of 4.27 h.

Here, we describe the development of a panel of VH4127 peptides conjugated in different manners to a siRNA targeting the ubiquitously expressed SOD1 mRNA. We evaluated the potential of these conjugates to bind to the LDLR and their functional delivery in both in vitro and in vivo studies.

## 2. Materials and Methods

### 2.1. Chemical Reagents and Material

Fmoc–amino acids were supplied from Iris Biotech (Marktredwitz, Germany). All other amino acids and reagents were purchased from Sigma-Aldrich (Saint-Quentin-Fallavier, France) or Analytical Lab (Castelnau-le-Lez, France). Peptide assembly was carried out using the Liberty (CEM^®^, Metthews, NC, USA) microwave synthesizer by solid phase peptide synthesis (SPPS) based on Fmoc/tBu strategy. Fmoc–Rink amide aminomethylpolystyrene resin (loading 0.74 mmol/g) and Fmoc–Gly-Wang resin (loading 0.66 mmol/g) were purchased from Iris Biotech and were used as solid support. All siRNAs were purchased from Horizon Discovery or Genelink.

### 2.2. Purification and Analytical Methods

#### 2.2.1. Peptide-Based Products

Monitoring of reactions and quality controls of the peptide-based intermediates were carried out by LC/MS using a Thermo Fisher Scientific UltiMate^®^3000 system (Waltham, MA, USA) equipped with an ion trap (LCQ Fleet) and an electrospray ionization source (positive ion mode). The LC flow was set to 2 mL/min using H_2_O 0.1%TFA (buffer A) and MeCN 0.1%TFA (buffer B) as eluents. The gradient elution was 10–90% B in 5 min (monitoring) or 10 min (quality control). The heated electrospray ionization source had a capillary temperature of 350 °C. Crude peptides were purified using reverse-phase (RP)-High Pressure Liquid Chromatography (HPLC) on a Thermo Fisher Scientific UltiMate^®^3000 system equipped with a C18 Luna™ (5 μm, 100 mm × 21.2 mm). Detection was assessed at 214 nm. The elution system was composed of H_2_O 0.1%TFA (buffer A) and MeCN 0.1%TFA (buffer B). Flow rate was 20 mL/min.

#### 2.2.2. Oligo-Based Products

Monitoring of reactions and quality controls of the intermediate and final products were carried out using HPLC-Mass Spectrometry (HPLC-MS) using a Thermo Fisher Scientific UltiMate^®^3000 system equipped with an ion trap (LCQ Fleet) and an electrospray ionization source (negative ion mode). The LC flow was set to 0.3 mL/min using HFIP 12.5 mM and DIEA 4 mM in H_2_O (buffer A) and HFIP 12.5 mM and DIEA 4 mM in MeOH (buffer B) as eluents. The gradient elution was 5–40% B in 18 min, and the column temperature was set at 65 °C. The heated electrospray ionization source had a capillary temperature of 350 °C.

### 2.3. Sequence and Modifications of siRNAs

siSOD1 sequences and modifications for this study were as follows: siSOD1 sense strand = 5′-P.mC.*.mA.*.mU.mU.mU.mU.2′-F-A.mA.2′-F-U.2′-F-C.2′-F-C.mU.mC.mA.mC.mU.mC.mU.mA.mA.mA.N6-3′, antisense strand = 5′-P.mU.*.2′-F-U.*.mU.mA.mG.2′-F-A.mG.2′-F-U.2′-F-G.mA.mG.mG.mA.2′-F-U.mU.2′-F-A.mA.mA.mA.mU.mG.*.mA.*.mG-3′, where mN and 2′-F-N represent 2′-O-methyl and 2′-Fluoro sugar-modified RNA nucleosides, respectively. P represents 5′-phosphate ending, VP represents 5′-vinylphopshonate, N6 represents the 6-carbon aliphatic arm finishing by an amine, and 5-LC-NU represents the modified nucleoside 5-aminohexylacrylamino-uridine bearing a 6-carbon aliphatic arm at the position 5 of the uridine. Finally, * represents a phosphonothioate linker (PS). All siRNAs were from Horizon Discovery (Cambridge, UK) and Genelink (Elmsford, NY, USA); they were desalted, and freeze-dried without further purification.

### 2.4. Preparation of Peptide Ligand Precursors

Peptides Pr-K(N_3_)-PEG2-[cMThzRLRGPen]c-NH_2_, Pr-[cMThzRLRGPen]c-PEG2-K(N_3_)-NH_2_, Pr-[cMThzRLRGPenG]c-OH, and Pr-K(N_3_)-PEG2-[cMThzRLRGPen]c-H_2_GH_2_GH_2_GH_2_-NH_2_, where “c” indicates a cyclic peptide, were synthesized using SPPS under microwave activation (Table 1). Briefly, Fmoc-Rink amide aminomethylpolystyrene for C-ter amide peptides or Fmoc–Gly-Wang resin for C-ter acid peptides were swollen in DMF for 10 min. Initial deprotection of the resin and stepwise assembly of the Fmoc–protected-amino-acids were performed under microwave activation using standard Fmoc/tBu peptide chemistry. Coupling times of 300 s were used with solution of Fmoc–amino acid (1 eq), Oxyma (10 eq excess; 1 M) in DMF, and DIC (5 eq excess; 1 M) in DMF under micro-wave activation at 70 °C. Fmoc removal was carried out with piperidine/DMF (20:80 *v*/*v*) for 200 s under micro-wave activation at 75 °C. Double coupling was used for Arg and Met amino acids to improve yield. In the special case of (D)-cysteine, coupling was performed at 50 °C for 360 s to avoid unwanted racemisation. Finally, the peptidyl-resin was washed successively with DCM, MeOH, and DCM. N-ter propionylation on solid support was carried out manually in a Pr_2_O/DCM (1:1) mixture for 5 min twice. The resin was further washed 3 times with DCm and then treated with TFA/TIS/H_2_O (95:2.5:2.5) containing DTT (100 mg/mL) at room temperature for 2 h. The cleavage solution was recovered, concentrated under N2 flow, and precipitated 3 times in cold diethyl ether. The crude products were dissolved in an H_2_O 0.1%TFA/MeCN 0.1% TFA (1:1) mixture and lyophilized. To form the disulfide bridge between the first D-Cys residue and the last Pen residue, peptides were dissolved in 0.5% aqueous AcOH (peptide concentration of 0.5 mg/mL). The pH of the peptide solution was adjusted to 8–9 with 2 M (NH_4_)_2_CO_3_, and K_3_Fe(CN)_6_ was added as a mild oxidative agent at room temperature for 0.5 h. The crude products were directly purified using preparative RP-HPLC as described previously. Final products were characterized to assess their purity and identity using HPLC-MS.

### 2.5. Preparation of siSOD1-Peptide Conjugates 

#### 2.5.1. siSOD1-Peptide Conjugate Synthesis Using SPAAC

The siSOD1 duplexes (1 eq) with a N6 or 5-LC-NU modification at the 3′-end of the sense strand were dissolved in an H_2_O/DMF (1:1) mixture to reach a concentration of 0.5 mM, in which DIEA (50 eq) was added. Then, DBCO-NHS (10 eq; 16.7 mM) was dissolved in DMF and added to the siSOD1 solution. The reaction was stirred at room temperature for 1.5 h and followed by RPLC-MS. When the reaction was complete, the siSOD1-DBCO was precipitated in 3 times the reaction volume of cold absolute ethanol (EtOH) and resuspended in water. Then, the peptide ligand containing an azidolysine (Pr-K(N_3_)-PEG2-[cMThzRLRGPen]_c_-NH_2_ or Pr-[cMThzRLRGPen]_c_-PEG2-K(N_3_)-NH_2_ or Pr-K(N_3_)-PEG2-[cMThzRLRGPen]_c_-H_2_GH_2_GH_2_GH_2_-NH_2_ was dissolved in a small volume of DMF and added to the siSOD1-DBCO solution under magnetic stirring for 1 h at room temperature followed by HPLC-MS until completion. The crude products were purified by precipitation in cold absolute EtOH or by filtration on Amicon 3 K, the final products were characterized using HPLC-MS, and finally quantified using optical density at 260 nm (Table 2).

#### 2.5.2. siSOD1-Peptide Conjugate Synthesis Using Direct Amidation

To a solution of peptide Pr-[cMThZRLRGPenG]c-OH in anhydrous DMF (20 eq; 1.68 mM) were successively added a solution of DIEA in anhydrous DMF (80 eq; 80.5 mM) and a solution of HATU in anhydrous DMF (20 eq; 20.16 mM) for pre-activation of the C-ter acid. Pre-activation was allowed to proceed at room temperature for 15 min and then at 40 °C for 5 min. Then, a solution of siSOD1-(3′SS)-N6 in H_2_O (1 eq; 6 mM) was added to the preactivated peptide and allowed to stir at 40 °C for 1 h. Monitoring of the reaction was performed using LC/MS. In the case of incomplete reaction after 1 h, a second addition of the preactivated peptide was performed in the same conditions, as described above. The crude products were purified using precipitation in cold absolute EtOH, the final products were characterized using HPLC-MS, and finally quantified using optical density at 260 nm (Table 2).

### 2.6. Surface Plasmon Resonance

His-tagged recombinant mouse LDLR (extracellular domain, mLDLR-ECD) was purchased from SinoBiological (Beijing, China). SPR measurements were performed at 25 °C using a Biacore T200 apparatus (Cytiva) and 50 mM HEPES-NaOH pH 7.4, 150 mM NaCl, 50 μM EDTA, 0.005% Tween-20 (*v*/*v*), 10 mM Imidazole as running buffer. mLDLR was immobilized on a NiHC1000 m sensor chip (Xantec, Dusseldorf, Germany) at a density of around 10–30 fmol/mm^2^. A control flowcell without mLDLR was used as reference. The multiple-cycle kinetic (MCK) method was applied to study molecular interactions of peptides and conjugates. Seven to 11 different concentrations of the analyte were prepared by two-fold dilutions in running buffer (0.6–640 nM or 0.6–40 nM) and successively injected over the flowcells during 120 s at 30 μL/min with a dissociation time of 300 s. Blank runs of running buffer were performed in the same conditions and subtracted from sample runs before evaluation. Equilibrium dissociation constants (K_D_) were calculated by plotting saturation binding curves using the equilibrium response at the plateau of all curves with BiaEvaluation version 2.0 software. The K_D_ values are summarized in Table 3. All data are the means of six determinations (triplicate analysis of 2 independent experiments).

### 2.7. Cell Culture and Reagents

Neuro-2a cells were provided from the European collection of authenticated cell cultures (ECACC) and cultured in DMEM High Glucose GlutaMAX™ (DMEM) supplemented with 10% fetal calf serum, 100 µg/mL streptomycin, and 100 U/mL penicillin in a humidified 5% CO_2_ atmosphere at 37 °C. Dubelcco’s Phosphate Buffer Saline (D-PBS), Phosphate Buffer Saline no calcium no magnesium (PBS), DMEM, fetal calf serum, Penicilin/Streptomycin, 0.05% Trypsin/EDTA (Trypsin), EDTA, DiI-LDL were purchased from Thermo Fisher Scientific.

### 2.8. In Vitro Validation of LDLR-Expressing Neuro-2a Cells

Neuro-2a cells were plated in a 96-well plate at 40,000 cells/well 2 days before experiments. Cells were incubated 3 h at 37 °C with red fluorescent DiI-LDL particles at 20 μg/mL, fluorescent cargo peptide A680-VH4127, or cargo scramble peptide A680-VH4Sc both at 1 µM, or co-incubated with DiI-LDL particles and A680-VH4127 or A680-VH4Sc at the same concentrations than previously described. Cells were extensively washed in D-PBS, then scrapped using Trypsin during 5 min at 37 °C and centrifuged for 5 min at 2000 rpm at 4 °C. Cells were fixed with PBS/EDTA 5 mM/paraformaldehyde 2% (PFA) for 15 min at room temperature. PFA was rinsed twice with PBS and removed using centrifugation as described previously, and cells were resuspended using with PBS/EDTA 5 mM before quantification of DiI and A680-associated fluorescence with Attune™ NxT flow cytometer equipped with Attune™ Cytometric software v5.2.0 (Thermo Fisher Scientific).

### 2.9. Cell Transfection

Neuro-2a cells were plated in a 96-well plate at 4000 cells/well one day before experiments. According to DharmaFECT general manufacturer’s protocol (Horizon Discovery), cells were transfected with a mix containing the DharmaFECT 2 and 30 nM of tested compound. This mix was incubated on cells in DMEM supplemented with 10% fetal calf serum during 24 h at 37 °C. At the end of the incubation period, Neuro-2a cells were washed once with D-PBS before RNA extraction.

### 2.10. Uptake and Free Uptake Gene-Silencing Experiments

Neuro-2a cells were plated in a 48-well plate at 13,000 cells/well the day before the test. All siRNA and conjugates were incubated during 3 days at 37 °C with 1 µM of conjugate prepared in DMEM supplemented with 1% fetal calf serum. At the end of the incubation period, cells were washed once with D-PBS before RNA extraction.

### 2.11. Animal Handling

Wild-type male C57Bl/6JRj mice (Janvier Labs, Le Genest-Saint-Isle, France) aged 10–12 weeks old were used. All animal studies were approved by the ethics committee for animal experimentation (CEEA-N°14) and approved by French ministry of agriculture (MRC). During all the experiments, animals were housed per groups of 4 animals, on a 12 h light/12 h dark cycle, with food and water access ad libitum. Intravenous (i.v., lateral tail vein) administrations (15 mg/kg) were performed on conscious restrained mice. Following a 7-day observational period, animals were euthanized by an intraperitoneal overdose of Ketamin-Xylasine mixture (Sigma Aldrich, Saint-Quentin-Fallavier, France), and tissue samples were collected after extensive blood wash-out by intracardiac perfusion (Left ventricle, Flow rate 8 mL/min) of a saline solution (0.9%) and immediately stored at −20 °C in 10 vol NucleoProtect RNA stabilization reagent (Macherey-Nagel, Düren, Germany) before further RNA extraction and target mRNA quantification.

### 2.12. RNA Extraction and cDNA Synthesis

For in vitro free uptake experiments, the total RNA was extracted using the NucleoSpin RNA XS (Macherey Nagel, Hoerdt, France) according to the manufacturer’s recommendations. For transfection experiments the SuperScript™ IV CellsDirect™ cDNA Synthesis kit (Thermo Fisher Scientific) was used according to manufacturer’s recommendations. For in vivo experiments, mouse organs were crushed in 2 mL tubes pre-filled with ceramic mixture in Precellys^®^Cryolys^®^ Evolution (Bertin, Montigny le Bretonneux, France) with a QIAZOL lysis buffer (Qiagen, Venlo, The Netherlands). A volume of 150 µL of chloroform (Sigma Aldrich) was added to 750 µL of tissue homogenate and a phenol/chloroform separation was performed using centrifugation for 15 min at 6000× *g* at 4 °C. Aqueous phases were recovered and RNA extraction was performed with the RNeasy 96 QIAcube HT Kit (Qiagen) according to the manufacturer’s recommendations, in a QIACUBE HT instrument (Qiagen). Quality and quantity of total RNA was determined with DNF-471 RNA Kit-15 nt (Agilent, Santa Clara, CA, USA) in a Fragment Analyzer 5300 (Agilent). For reverse transcription (RT), 500 ng of total RNA were used (except for cells treated with SuperScript™ IV CellsDirect™ cDNA Synthesis), and cDNA synthesis was performed using the High-Capacity RNA-to-cDNA™ kit (Thermo Fisher Scientific) according to the manufacturer’s recommendations.

### 2.13. Real-Time Quantitative PCR

Real-time quantitative PCR (RT-qPCR) assays were performed using the CFX96 Touch Real-Time PCR Detection System (Bio-Rad, Hercules, CA, USA). Amplifications were carried out in a 10 μL final reaction solution containing 12.5 ng of cDNA, 1× of TaqMan™ Fast Universal PCR Master Mix), 1× of TaqMan^®^ Gene Expression Assay Mix (Thermo Fisher Scientific), and RNase-free water, according to the manufacturer’s recommendations. The following primers were used: Mm01344233_g1 SOD1 (mouse), Mm02526700_g1 RpL13 (mouse), Mm01352366_m1 SDHA (mouse), Mm00457191_m1 PSMC4 (mouse). RpL13, SDHA, or PSMC4 served as internal controls for sample normalization, and the comparative cycle threshold method (2−ΔΔCt) was used for data quantification. Finally, gene expression ratios (compared to control samples) were determined.

### 2.14. Data Analysis

Statistical comparison of the knock-down (KD) effect of tested molecules in both in vitro free uptake and in vivo experiments was performed using a one-way ANOVA followed by a Dunnett’s multiple comparisons test. In vivo experiments were performed blind from treatment administration until data analysis and freezing (all formulations and tissue samples were coded).

## 3. Results

### 3.1. Molecular Design and Synthesis of siSOD1-Peptide Conjugates

**Figure 1:**
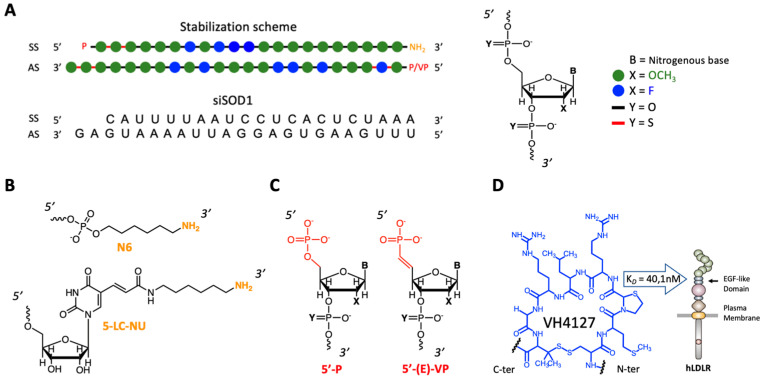
General design of siSOD1-peptide conjugates. (**A**) Detail of the stabilization scheme of siSOD1 duplex. Chemical modifications: green dot = 2’-Ome; blue dot = 2’-F; red line = PS; P = phosphate; VP = vinylphosphonate. (**B**) Detailed structure of the reactive amine at the 3′-end of the sense strand (SS) for further conjugation. N6 = 6-carbon aliphatic arm ending with an amine group. 5-LC-NU = 5-Aminohexylacrylamino-Uridine, modified uridine with an aminohexylacrylamine arm at position 5. (**C**) Detailed structure of the 5′-end of the antisens strand (AS). P = standard phosphate; (E)-VP = modified and metabolically stable vinylphosphonate with a double bond in E configuration. (**D**) Detail of the VH4127 peptide sequence (cyclo[(D)-Cys-Met-Thz-Arg-Leu-Arg-Gly-Pen]) and scheme of its structure. Disulfide cyclization occurred between the penicillamine and cysteine side chains. VH4127 binding affinity to LDLR: Kd = 40.1 nM, surface plasmon resonance (SPR): Biochip NiHC1000m; mode MCK).

The design of the siSOD1-peptide conjugates involved three-partners (Figure 1), namely: (i) the peptide ligand allowing specific tissue-targeting; (ii) the pharmacologically active siRNA moiety; and (iii) the linker that is essential for linking these two functional entities while retaining their biological functions.

**Figure 2:**
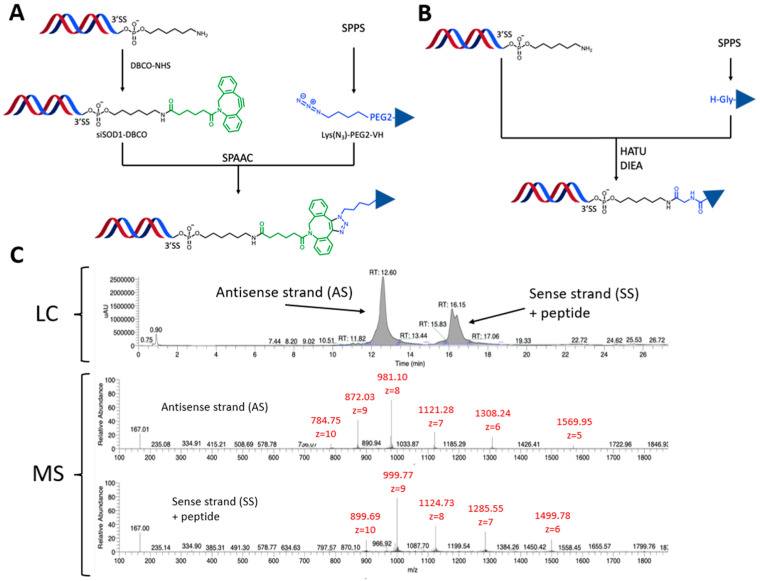
Synthesis strategies and characterization methods of siSOD1-peptide conjugates. (**A**) Production process of siSOD1-peptide conjugates. A constraint alkyne (DBCO) was introduced on the N6 modification at the 3′ end of siSOD1 sense strand. An azide function was incorporated in the VH4127 peptide sequence during SPPS in the form of azidolysine (Lys(N_3_) or K(N_3_)) and spaced from it with a PEG2. (**B**) Alternative synthesis strategy of siSOD1-peptide conjugates. The siSOD1-peptide conjugate was obtained through direct amidation between the N6 modification at the 3′ end of siSOD1m sense strand and the free carboxylic acid of peptide VH4127. (**C**) LC/MS characterization of siSOD1-peptide conjugates. Buffer A: HFIP 12.5 mM and DIEA 4 mM in H_2_O; Buffer B: HFIP 12.5 mM and DIEA 4 mM in MeOH. Flow rate was 0.3 mL/min and column temperature set at 65 °C. Detection was performed at 260 and 214 nm. MS analysis in negative mode and spectra was deconvoluted.

Since the VH4127 peptide was previously selected, optimized, and validated for its ability to preferentially distribute in vivo to LDLR-enriched tissues [28,29], the present work focused on the siRNA and the chemistry process used for its conjugation to the VH4127 peptide. We explored different conjugation strategies to evaluate the impact of the different designs on the physico-chemical properties of the conjugates and on their in vitro and in vivo efficacies. Two different methodologies were investigated: (i) an indirect convergent strategy through a strain-promoted azide alkyne cycloaddition (SPAAC) process that requires the first parallel introduction of suitable moieties on both the siSOD1 and the peptide for further click conjugation (Figure 2A,B); and (ii) a direct conjugation by an amide bond that does not require prior functionalization of the siRNA and hence, the introduction of a linker. A murine siSOD1 was studied in this work based on the stabilization scheme described by Foster et al. [31]. It encompasses chemical modifications that increase its resistance to nucleases and silencing potency while diminishing potential off-target effects and cytotoxicity. The duplex was composed of a sense strand (SS) of 21 nucleotides, with an hexylamino (N6) modification at the 3′-end, and an antisense strand (AS) of 23 nucleotides with a 2 nucleotide 3′-overhang ending. The N6 modification is a 6-carbon aliphatic arm ending with a reactive primary amine or a 5-LC-NU modification for further chemical functionalization. Finally, both SS and AS sequences contained a 5′-modification, respectively, a 5′-phosphate or 5′-vinylphosphonate modification (Figure 1B,C). Among the different bioconjugation strategies, the SPAAC or copper-free “Click-Chemistry” reaction is particularly suitable for post-synthetic and site-specific conjugation of large biomolecules such as siRNAs [32,33]. Indeed, this reaction presents several advantages: (i) the reactive functions involved are inert towards other chemical functions, conferring major reaction selectivity; (ii) the reaction takes place at room temperature in both aqueous and organic solvents, thus facilitating the solubilization of reagents; and (iii), in opposition with the first version of this reaction, there is no need for copper as a catalyst, which is cytotoxic and particularly difficult to extract from the final product.

To prepare the siSOD1-peptide conjugates based on the above-mentioned click strategy, we chose to introduce the azide moiety onto the peptide and the alkyne moiety onto the siRNA. With this aim, an unnatural azidolysine bearing an azide function was added to the VH4127 peptide, either at its C-ter or N-ter during the SPPS. To retain the affinity of the VH4127 peptide towards the LDLR, the azidolysine was spaced out from the bulky cyclic peptide by introduction of a PEG2 linker. As for the constrained alkyne function, it was introduced post-synthetically using a reaction of a heterobifunctional linker DBCO-NHS with the amine group of the hexylamino (N6) modification. The final click conjugation step allowed covalent attachment of the azido-peptide to the siSOD1-DBCO to obtain the siSOD1-peptide conjugate using SPAAC (Figure 2A). Additionally, to investigate the impact of the conjugate design on its biological properties, a direct conjugation method was investigated: the coupling without prior addition of the DBCO-NHS linker was achieved by a straightforward amidation between the siRNA’s N6 modification and the VH4127 peptide through its carboxylic acid C-ter (Figure 2B).

Cationic and histidine-rich peptides can facilitate endosomal escape. For this reason, the H8G3 poly-His stretch was introduced in C-ter of the VH4127 peptide. We have internal evidence that the H8G3 poly-His stretch in another context does significantly improve the cellular accumulation of the H8G3 functionalized VH4127. However, in the present study, we did not observe an improved KD effect of siSOD1-34 that includes this H8G3 poly-His stretch when compared to the structurally similar siSOD1-31 conjugate. One hypothesis is that the siRNA cargo conjugated to VH4127-H8G3 hampers the expected endosomal escape properties of the H8G3 poly-His stretch. siRNAs are very peculiar compounds from a physico-chemical point of view. They could interfere with VH4127-H8G3 and, as a consequence, limit the endosomal properties of the H8G3 poly-His stretch.

All crude conjugates were purified using HPLC and characterized with HPLC-MS. Molecular mass of both antisense and sense strands was calculated using manual deconvolution. The HPLC conditions allowed separation of the two strands on columns and consequently separate ionization in MS. All HPLC-MS analysis of siSOD1-peptide conjugates showed, for the sense strand, a double peak (UV) with the exact same mass. This double peak resulted from the formation of two regioisomers during the click-chemistry reaction step between the DBCO group and the azidolysine [31] (Figure 2C).

### 3.2. LDLR-Binding Affinity of siSOD1-Peptide Conjugates Using Surface Plasmon Resonance (SPR)

Chemical design of the conjugates may directly impact the affinity of the conjugates and thus modulate their productive uptake, intracellular trafficking, and, therefore, the biological or therapeutic effect [17,34] of siRNA-peptide conjugates. We thus prepared a panel of different conjugates to investigate different parameters in the siSOD1 conjugate chemistry (synthesis scheme Figure 2; characterization data, Table 3; SPR raw data, Supplemental Figure S1). In general, the peptide conjugation at the 3′-end of the siSOD1 sense strand impacted only moderately its LDLR-binding affinity, with Kd values ranging from c.a. 10 to 100 nM, compared to the VH4127 peptide ligand alone with a Kd of 40 nM. While the siSOD1-33, -35, -36, -37 conjugates demonstrated the highest LDLR-binding affinity, the siSOD1-31 and -32 conjugates showed a slightly lower Kd. Notably, we observed that the presence of the 5′VP on the siSOD1-36 conjugate positively influenced receptor/ligand interaction in comparison with the siSOD1-32 conjugate, its homologue without the 5′VP modification. Interestingly, this was not observed with the siSOD1-37 and siSOD1-33 conjugates, homologues with and without 5′VP, respectively, that exhibit LDLR-binding affinities in the same range. Peptide conjugation on the 5-LC-NU modification in these two conjugates, originally used to prevent interactions between a double-strand oligonucleotide and any large cargo linked to it, may explain this similarity by favoring ligand presentation on its target. Unfortunately, the siSOD1-34 conjugate could not be explored using SPR due to the strong complexation of the histidines contained in its structure with the nickel present on the SPR chips. Once confirmed that all our conjugates retained their binding affinity for LDLR, we next explored their gene-silencing efficacy in vitro on a Neuro2-A cell line.

### 3.3. Gene-Silencing Potency of siSOD1-Peptide Conjugates In Vitro in Murine Neuro-2A Cells

**Figure 3:**
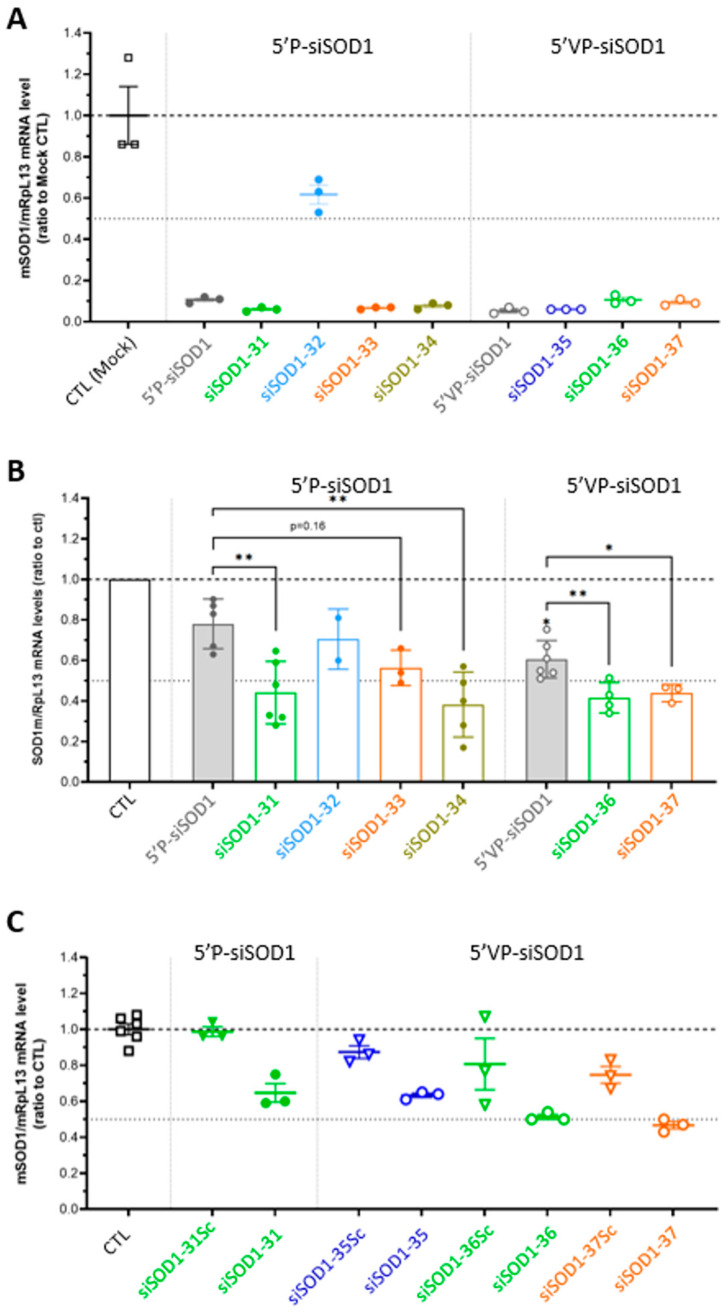
Gene-silencing potency of unconjugated siRNAs (5′P-siSOD1 and 5′VP-siSOD1) and conjugated siRNAs (siSOD1-31, -32, -33, -34, -35, -36, -37) using transfection and free uptake. (**A**) Transfection of unconjugated siSOD1 and siSOD1-peptide conjugates on Neuro-2a cells at 30 nM. After 24 h of incubation mSOD1 mRNA levels were quantified using RT-qPCR. (**B**) Free uptake experiments of unconjugated siSOD1 and siSOD1-peptide conjugates on Neuro-2a cells at 1 µM. After 3 days at 37 °C, mSOD1 mRNA levels were quantified using RT-qPCR. Each dot corresponds to the mean value obtained in independent experiments. (**C**) Free uptake experiments of siSOD1-31, -35, -36, -37 conjugates with their respective negative controls siSOD1-31Sc, -35Sc, -36Sc, -37Sc on Neuro-2a cells at 1 µM. After 3 days at 37 °C, mSOD1 mRNA levels were quantified using RT-qPCR. * *p* < 0.05; ** *p* < 0.01.

The in vitro gene silencing potential of siSOD1-peptide conjugates was investigated in the murine Neuro-2A (N2A) cell line after lipofection to verify their intrinsic RNAi activity, or free uptake to evaluate their potential to undergo LDLR-mediated functional uptake, leading to KD of the mSOD1 target mRNA. The ability of both LDLR ligands, including LDL particles (DiI-LDL) and the previously described LDLR-binding VH4127-A680 conjugate (vs. its non-binding scrambled version VH4sc-A680) [35] to specifically bind the murine LDLR (mLDLR) expressed by N2A cells, was verified beforehand (Supplemental Figure S2). First, transfection experiments clearly demonstrated that most of the tested siSOD1-peptide conjugates and the unconjugated siSOD1 induced similar KD effects (c.a. 90%) (Figure 3A). The only exception was the siSOD1-32 conjugate (SPAAC with siRNA-N6 conjugation on the C-ter of the peptide) with a KD potential of 40% following transfection. Second, free uptake experiments consistently demonstrated higher KD effect, up to ~60%, for most of the conjugates tested, compared to unconjugated siSOD1 molecules, demonstrating a higher functional uptake potential for LDLR-binding conjugates (Figure 3B). Interestingly, whereas the unconjugated 5′VP-siSOD1 showed a slightly higher KD potential in this cellular model than the 5′P-siSOD1, with a mean of 40% vs. 22%, respectively, this did not translate into a higher KD potential of 5′VP-siSOD1-peptide conjugates in the in vitro system. Again, the only exception among siSOD1-peptide conjugates was the siSOD1-32 conjugate, which did not show improvement compared to the unconjugated 5′P-siSOD1, consistent with its lower KD potential using lipofection. The results obtained with the 5′P-siSOD1-peptide conjugates also showed that the addition of a H8G3 poly-His stretch in C-ter of the VH4127 peptide, introduced to potentially increase endosomal escape [36], did not translate into an improved KD effect, as compared to the structurally similar siSOD1-31 conjugate. Although we cannot rule out a higher dissociation from LDLR in early/sorting endosomes and thereby a higher delivery to late compartments, this may not result in higher endosomal escape and delivery to the cytosol. All the conjugates encompassing the non-binding scrambled peptide rather than the VH4127 peptide elicited only low KD effects, thus confirming the prominent role of the VH4127 peptide and LDLR in the functional uptake and KD effect of our siSOD1-peptide conjugates (Figure 3C). Finally, the minor KD effect of all tested conjugates comprising the non-binding scrambled peptide confirmed the involvement of LDLR in the functional uptake and KD effect of our siSOD1-peptide conjugates (Figure 3C). We also investigated an additional conjugate where the VH4127 peptide was directly conjugated to the amino group of the 5′P-siSOD1-N6 precursor, leading to a conjugate with a much smaller linker. As observed with other conjugates, the resulting siSOD1-35 conjugate showed an improved KD effect compared to its non-binding control siSOD1-35sc. Altogether, these results prompted us to further investigate in vivo the KD potential of the LDLR-binding conjugates.

### 3.4. Gene-Silencing Potency of siSOD1-Peptide Conjugates after Systemic Administration in Mice

**Figure 4:**
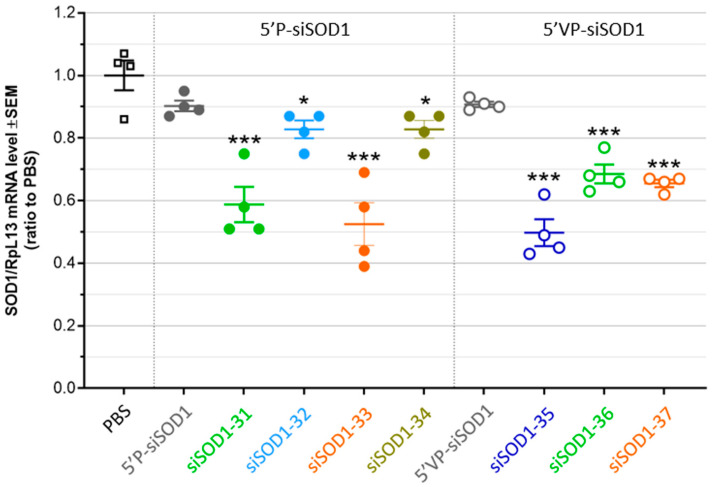
In vivo gene-silencing potency of unconjugated siRNAs (5′P-siSOD1 and 5′VP-siSOD1) and conjugated siRNAs (siSOD1-31, -32, -33, -34, -35, -36, -37) in mice liver. Mice were injected (i.v. lateral tail vein) with unconjugated siRNAs and siSOD1-peptide conjugates in PBS (15 mg/kg). Seven days post-administration mice were euthanized and perfused with saline solution (0.9% NaCl). Organs were collected to assess the SOD1 mRNA levels using RT-qPCR. Each dot corresponds to one mouse. * *p* < 0.05; *** *p* < 0.001.

The in vivo targeting and functional uptake potential of LDLR-binding siSOD1-peptide conjugates was evaluated as follows. Seven days after single intravenous (i.v. bolus) administration in mice at 1 µmole/kg (corresponding to 15 mg/kg siRNA), SOD1 mRNA expression level was quantified using RT-qPCR in the liver, an organ expressing high levels of the target LDL receptor and where the VH4127 peptide previously demonstrated efficient distribution [28,29]. As expected, both the unconjugated siSOD1 showed poor KD effect, even in the presence of the stabilizing 5′VP-AS modification (~10% KD, ns) (Figure 4). On the contrary, siSOD1 conjugation to the LDLR-targeting VH4127 peptide consistently led to a significant KD effect, reaching c.a. 50% KD with both siSOD1-33 and -35 conjugates. No clear correlation could be evidenced between in vitro free uptake and in vivo KD results. First, introduction of the 5′VP-AS modification in the unconjugated siSOD1, which was shown to increase both AS metabolic stability and RISC-engagement [37,38], did not improve the liver KD effect, as observed during our in vitro free uptake experiments (Figure 3B). Second, besides the siSOD1-32 conjugate that showed only a minor yet significant KD effect (~20%, *p* < 0.05) as observed in free uptake experiments, the siSOD1-33 and siSOD1-35 conjugates with a 5′P-AS and a 5′VP-AS, respectively, both demonstrated the highest KD potential, with a c.a. 50% reduction in mSOD1 mRNA levels (*p* < 0.001), while the siSOD1-31, -34, -36, and -37 conjugates performed best in our in vitro setting.

## 4. Discussion and Perspectives

Many attempts have been made this last decade to overcome the inherent barriers to the clinical use of therapeutic siRNAs and establish the necessary foundations for the development of strategies that allow their functional delivery to a tissue of interest. With more than 30 years of efforts on the chemistry and stabilization of siRNAs, it is now possible to produce molecules containing chemical modifications to achieve high metabolic stability, high target sequence specificity, and efficacy. Nevertheless, exploiting the full clinical potential of siRNAs [39] still requires the development of efficient systems to address major delivery hurdles including fast plasma clearance, low tissue selectivity, and low functional uptake in target cells. In the present work, we validated the potential of a previously described LDLR-binding cyclic peptide [28,29,35] for the in vitro and in vivo targeting and productive delivery of therapeutic siRNAs. Although no clear in-vitro-to-in-vivo structure-activity relationship arose for our LDLR-targeting siSOD1-peptide conjugates, as discussed thereafter, it clearly appeared that their LDLR-binding potential translates into an active functional uptake in cells in vitro and in vivo in the liver, leading to significant RISC engagement and KD effect.

As shown in an early study by Gilleron et al., where small molecules and different delivery systems based on either LNPs or molecular cholesterol-siGFP conjugates were screened for their functional uptake potential, improvement of gene silencing occurred on either the uptake system per se, or on subsequent trafficking and endosomal escape steps [40]. RNAi induced by siRNA-ligand conjugates occurs in four basic steps: (i) the effective conjugate recognition by the targeted receptor, without affecting the endogenous ligand interaction; (ii) the endocytosis of the siRNA-ligand/receptor complex within cells, followed by conjugate dissociation from receptors into early and/or sorting endosome; (iii) the siRNA endosomal escape to reach the cytoplasmic compartment; and (iv) its recognition and loading into the RISC complex.

In the present study, we explored the potential of LDLR-binding peptides to efficiently transport siRNA into cells, and to evaluate whether the modulation of some of the parameters of siRNA-peptide conjugates could impact their efficacy. The LDLR represents an attractive cell-surface target receptor to support efficient functional delivery of therapeutic oligonucleotides into different cells and organs, owing to its relatively high cell surface expression, differential expression levels in different organs, high level expression in some tumors, and its ability to undergo hundreds of endocytosis cycles during its 20 h lifespan [24,29,41,42]. However, it is worth noting that efficient functional delivery of an anti-sens oligonucleotide (ASO) to beta pancreatic cells in mice could be achieved by means of a glucagon-like peptide-1 (GLP-1) synthetic peptide ligand targeting the GLP1 receptor (GLP1R), a cell-surface receptor from the G protein coupled receptors (GPCRs), known for its rather low expression and low endocytic capacity [43,44]. Therefore, beyond the initial assumption that delivering therapeutic oligonucleotides to extrahepatic sites requires new Ligand/Receptor pairs displaying features similar to those of the prototypical GalNAc/ASGPR pair, it appears that other parameters can largely compensate for poor expression and endocytic potential. In line with this, the molecular mechanisms underlying and enabling the mostly inefficient (below 1%) transit of Ligand–Oligo conjugates from early endocytic vesicles to the cytosol or nucleus, where the oligo can eventually engage its target, remain poorly understood [45,46,47,48]. For these reasons, and given the unique features of each new ligand/receptor pair, investigation of the structure–activity relationship underlying Ligand–Oligo activity still remains rather empirical [49]. In the present work, the in vitro studies allowed us in the first place to assess the efficacy of different conjugates and to identify those that fitted our critical criteria: (i) binding affinity for the hLDLR and (ii) siRNA knockdown efficacy. All conjugates tested demonstrated significant KD by free uptake. Hence, in a second place, we further investigated them in the more complex in vivo setting where ADME (Absorption, Distribution, Metabolism, and Excretion) parameters may impact the tissue exposure, functional uptake, and KD efficacy of each ligand–siRNA conjugate.

A siSOD1 was studied based on the stabilization scheme described by Foster et al. [31]. It encompasses chemical modifications that increased its resistance to nucleases and silencing potency while diminishing potential off-target effects and cytotoxicity [31]. We selected the N6 and the 5-LC-NU modifications as reactive functional amino groups on the 3′ end of the sense strand for further conjugation with our peptide ligands. Antisense strand 5′end modifications are also known to increase siRNA stability and potentiate their effects presumably via enhanced loading within the RISC complex. Thus, we also compared conjugates encompassing a 5′-phosphate or 5′-vinylphosphonate modification on the siRNA antisense strand.

From a general medicinal chemistry perspective, conjugation modality and linker chemistry used so far in recent Ligand–Oligo conjugates generally involve either direct amidation coupling or SPACC. Examples of these conjugation strategies include siRNA–GalNac conjugates [50], ASO-peptide conjugates [44,49,51], siRNA–antibody conjugates [52]. SPAAC offers high coupling yields and versatility, allowing linkage of complex biomolecules in a highly specific manner. Nonetheless, the bulky and hydrophobic nature of the constrained alkyne precursor (DBCO, BCN) can possibly influence the conjugate structure, and thus negatively impact its physico-chemical proprieties. On the contrary, direct amidation coupling allows a more straightforward coupling and generates a much smaller, natural, hydrophile, and flexible linker. However, this coupling strategy is less versatile and requires two highly reactive chemical partners, namely a primary amine and a carboxylic acid, and warrants careful upstream considerations on the synthesis strategy.

Considering the bulky and charged nature of our peptide ligand, consisting of a constrained cyclic octapeptide composed of two arginine residues, and its use as a targeting ligand for a covalently attached siRNA, the modulation of its anchoring site, as well as the linker length and nature, can have a profound impact on its orientation and accessibility for optimal binding to the LDLR. In the present work, different siSOD1-peptide conjugates were generated with the objective of investigating how these parameters can impact LDLR-binding and siSOD1-peptide KD potential in both a cellular model and after systemic administration in mice. Interestingly, with respect to their LDLR-binding potential, our results show rather low impact of conjugation site and chemistry on Kd values compared to the unconjugated peptide (40 nM), demonstrating a very permissive peptide/LDLR interaction even in the presence of the conjugated siRNA. Variation of parameters such as (i) the peptide coupling site, (ii) the sense strand 3′-end coupling site with the 5-LC-NU modification (Figure 1B), or (iii) the linker length and nature, i.e., much smaller and less hydrophobic, produced by direct coupling (Figure 2B), did not induce drastic increase or loss of LDLR-binding affinity. Indeed, the affinities of most conjugates remained in the range of 11 to 32 nM as compared to the unconjugated reference peptide. Surprisingly siSOD1-31 and siSOD1-32 showed slightly decreased affinity for the LDLR, in the range of 87–104 nM. However, all conjugates were validated for the in vitro and in vivo studies.

Therefore, one can anticipate broad application potential of our LDLR-targeting ligands in terms of oligonucleotide modality, such as gapmer ASOs, ssASOs, etc. On the contrary, other ligand/receptor pairs investigated for oligonucleotide-targeted delivery purposes demonstrated altered binding and uptake by their target receptor that could be attributed to interference between the negatively charged oligonucleotide backbone and positive charges of the ligand. These include the neurotensin (NT) peptide proposed as a targeting ligand for improved delivery to neurons expressing the neurotensin-receptor [53], or the GLP1 that targets the GLP1R expressed in pancreatic beta cells [44,49].

After demonstrating adequate binding of our siSOD1-peptide conjugates to cell-surface LDLR, the different coupling strategies proposed in the present work could impact downstream events. One of the most critical steps following receptor-mediated endocytosis relies on peptide/LDLR dissociation in sorting endosomes and subsequent creation of a late endosomal or lysosomal depot, a prerequisite for siRNAs to slowly egress in the cytosol where they can engage the RISC. We could not verify the efficient uptake and intracellular accumulation of the siSOD1-VH4127 conjugates of the present study. However, using a structurally similar and fluorescent A680-VH4127(3′SS)-siGFP conjugate, we have evidence that it efficiently transitioned from LDLR-positive early compartments soon after uptake, to LDLR-negative late compartments after longer incubation times. As performed by most studies in the field, these intracellular trafficking steps were investigated indirectly, by measuring the final functional read-out of our SOD1-peptide conjugates, namely the mSOD1 mRNA KD, in both a cellular setting and after systemic administration in mice. Importantly, our results clearly demonstrate a significant improvement of siSOD1 functional uptake and gene KD, when conjugated to the LDLR-targeting VH4127 peptide, both in murine Neuro-2a cells expressing functional LDLR and in vivo in the liver of mice injected with our conjugates, with c.a. 50% reduction in mSOD1 mRNA levels with the best conjugates, while only minor effects could be observed with the unconjugated siSOD1. These results thus demonstrate that the LDLR-binding peptide not only maintains its binding potential when conjugated to a siRNA, but also mediates efficient uptake leading to significant gene KD both in vitro and in vivo as summarized in Table 4. However, we observed a discrepancy in the relationship between the conjugation strategy and the KD efficiency in either our cellular model or in mice. The best performing conjugates after systemic administration in mice were the siSOD1-33 and the siSOD1-35 conjugates. In the siSOD1-33 conjugate, the VH4127 peptide is coupled via an N-ter Lys(azido)-PEG2- group to a pseudo-nucleotide (5-LC-NU) extending from the 3′-end of the sense strand of a 5′P(AS)-siSOD1. The siSOD1-35 conjugate was obtained using direct coupling of the VH4127 via a C-ter-Gly-OH group to an hexylamino group protruding from the 3′-end of the sense strand of a 5′VP(AS)-siSOD1. Surprisingly, these two conjugates showed rather low functional uptake potential upon free uptake in Neuro-2a cells compared to other LDLR-binding conjugates. One hypothesis could be that different cellular exposure durations between in vitro and in vivo setting, likely much shorter in vivo compared to the continuous 3-day exposure in our cellular model, may lead to differential intracellular accumulation and KD effects between our different conjugate designs. Second, liver hepatocytes in vivo vs. cultured murine neuroblastoma cells can display profound differences in their intracellular dynamics, making some molecular designs more prone to reaching the cytosolic RISC complex in pharmacological amounts. Although the use of freshly collected mouse liver hepatocytes might represent a more relevant in vitro model, our screening approach was based on the use of a stable cell line to ensure higher robustness across experiments while limiting the use of in vivo material.

Recent studies showed that siRNA accumulation and stability in acidic intracellular compartments is critical for long-term activity [54], and that fewer than 1% of endocytosed molecules reach the cytosol compartment [23,43,45,47,55]. Therefore, beyond the identification of new ligand/receptor pairs able to support efficient functional uptake of therapeutic oligonucleotides, endosomal escape remains a rate-limiting step for oligonucleotide functional delivery, and there is a high need to investigate new strategies to increase the amount of molecules reaching the cytosol [56]. Although several methods have been investigated, based on either osmolytic compounds such as chloroquine or membrane-destabilizing agents such as peptides derived from the influenza hemagglutinin (HA), they all show toxicity making them non-viable approaches for in vivo applications [48,54,56,57]. In the present work, we investigated an original approach by introducing a pH-conditional endosomal escape-inducing peptide (EEIP) directly conjugated in C-ter of our VH4127 peptide. Because the imidazole group of histidines have a pK of 6.0, corresponding to the pH found in early and sorting endosomes, they can remain neutral at extracellular pH while being protonated when reaching these endosomal compartments following receptor-mediated endocytosis. Several studies have shown that poly-histidine stretches can act as EEIPs by translocation across the endosomal membrane, thereby improving the KD potential of the conjugated siRNA [36,58]. We have evidence that conjugation of a 8-histidine stretch in C-ter of our LDLR-binding VH4127 peptide led to a strong increase in intracellular delivery in different in vitro cellular models, and that this translates into a 2-to 3-fold increase in LDLR-enriched tissue exposure after systemic administration in mice. In the present study, the siSOD1-34 conjugate encompassing the same linear 8-histidine stretch in C-ter of the VH4127 peptide was produced to explore its potential to increase the functional delivery of the siRNA. Unfortunately, the LDLR-binding affinity of this conjugate could not be evaluated using SPR due to the strong complexation of histidine with the nickel present on the chips. Despite promising results in transfection, this conjugate did not show higher KD effect in vitro and even showed a lower KD effect in vivo. As suggested from positively-charged peptide ligands or cell-penetrating peptides (CPPs) [53,56], unwanted molecular interactions occurring in sorting and late endosomes between the positively charged poly-histidine moiety and the negatively charged siRNA backbone could mask the protonated poly-His stretch. This could in turn hamper the expected benefit in late endosomal delivery and/or endosomal escape potential, which might explain the observed drop in efficacy [55].

Finally, even if the conjugates enter cells and undergo endosomal escape, their ability to load into the RISC depends on the presence of a phosphate group at the 5′-end of the active/antisense strand [59]. Because this functionally crucial moiety might be cleaved by lysosomal phosphatases, introduction of the 5′VP modification represents a suitable alternative to 5′P, with a potential to improve the silencing effect of our conjugates [37,38]. The siSOD1-35, -36, and -37 conjugates were produced with a 5′VP instead of a 5′P to investigate this aspect. None of these conjugates showed a higher KD effect than with the 5′P-containing conjugates, both in free uptake experiments and in vivo. One possible explanation is that the expected benefit from this 5′VP modification, namely higher metabolic resistance in lysosomal compartments, cannot occur due to insufficient delivery to these compartments. In this hypothesis, the rate-limiting step of the LDLR-binding siSOD1-peptide conjugates might rely on an earlier step during intracellular trafficking, such as low dissociation from LDLR in sorting endosomes leading to recycling back to the cell surface (non-productive uptake).

The present work demonstrates that our LDLR-targeting peptides can support efficient functional uptake of a model siRNA in both in vitro and in vivo settings. However, we need to gain further understanding on the rate-limiting steps and identify strategies to further improve the conjugate design to obtain significant KD effects at lower concentrations and doses. One possible approach includes the use of a fluorescent probe, such as A680, conjugated at the 3′-end of the active/antisense strand, allowing direct tracking in cellular models and evaluation of the intracellular fate of tested conjugates. We previously used this approach to visualize early recycling vs. lysosomal trafficking pathways and quantify the cellular elimination profile of fluorescent conjugates [35]. Another strategy relies on the use of GalNAc conjugated to the fusogenic peptide INF7, as performed previously [54], to force endosomal escape of potential intra-vesicular depot of an oligonucleotide in liver hepatocytes, if any. In this approach, any improvement of the KD effect following GalNAc-INF7 injection would confirm that the oligonucleotide was efficiently delivered in cells, but with endosomal escape as the rate-limiting step towards optimal RISC-engagement and KD effect. On the contrary, no improvement would indicate insufficient intravesicular depot, and could guide further design optimization towards higher uptake and dissociation from the LDLR in early/sorting endosomes.

## 5. Conclusions

We validated previously described synthetic LDLR-binding peptides, including the VH4127 peptide, as viable ligands able to trigger efficient LDLR-mediated functional delivery of therapeutic oligonucleotides, both in a cellular model and in vivo after systemic administration in mice. Importantly, our results clearly demonstrate efficient mRNA KD (c.a. 50%) of some of the siRNA-peptide conjugates we developed at a given time point. The KD effects may differ across time and can certainly be further improved to reach near complete KD by modulating siRNA-Ligand conjugation, administered dose, SC vs. IV route of administration, and may differ depending on organs (liver, duodenum, adrenal medulla, etc.) and diseased tissues (different tumors). Although no clear in-vitro- to-in-vivo activity correlation could be made, our results highlight several potential tracks for further optimization of this new class of Ligand–Oligo conjugates. Since the LDLR is differentially expressed in organs and is overexpressed in many cancers [24,29,41,42], the present work opens new opportunities and warrants further evaluation for oligonucleotide delivery to different organs or in tumors that otherwise do not support functional uptake of naked oligonucleotides.

## Figures and Tables

**Table 1:** LC/MS characterization of peptide ligand precursors.

Peptide Sequences	RP-HPLC, Retention Time (min)	HPLC-UV Purity (%)	Theoretical Mass (Da)	ESI-MS (m/z) [M,H]^+^ (*Calculated*)
Pr-K(N_3_)-PEG2-[cMThzRLRGPen]_c_-NH_2_	rt = 3.36 min	91.7%	1332.60 Da	1333.51 Da
Pr-[cMThzRLRGPen]_c_-PEG2-K(N_3_)-NH_2_	rt = 3.61 min	96.7%	1332.60 Da	1333.00 Da
Pr-[cMThzRLRGPenG]_c_-OH	rt = 2.90 min	97.0%	1091.45 Da	1092.69 Da
Pr-K(N_3_)-PEG2-[cMThzRLRGPen]_c_-H_2_GH_2_GH_2_GH_2_-NH_2_	rt = 2.68 min	90.5%	2244.95 Da	2245.44 Da

**Table 2:** LC/MS characterizations of siSOD1-peptide conjugates.

	Sense Strand + Peptide			Antisense Strand		
	RP-HPLC, Retention Time Gradient	Theoretical Molecular Weight	ESI-MS (m/z)	RP-HPLC, Retention Time Gradient	Theoretical Molecular Weight	ESI-MS (m/z)
siSOD1-31	16.32 min 16.54 min	8709.66 Da	8710.80 Da 8710.65 Da	12.64 min	7855.17 Da	7855.73 Da
siSOD1-31Sc	14.18 min 14.33 min	8662.56 Da	8663.22 Da 8663.03 Da	11.44 min	7855.17 Da	7854.96 Da
siSOD1-32	15.45 min 15.59 min	8709.66	8709.80 Da 8709.60 Da	12.24 min	7855.17 Da	7855.70 Da
siSOD1-33	16.15 min 16.40 min	9004.91 Da	9005.42 Da 9005.29 Da	12.60 min	7855.17 Da	7856.04 Da
siSOD1-34	16.30 min 16.50 min	9977.95 Da	9978.06 Da 9978.08 Da	12.64 min	7855.17 Da	7856.19 Da
siSOD1-35	9.30 min	8166.97 Da	8166.66 Da	7.99 min	7851.18 Da	7850.70 Da
siSOD1-35Sc	8.12 min	8088.89 Da	8088.74 da	8.03 min	7851.18 Da	7850.79 Da
siSOD1-36	15.47 min 15.64 min	8709.66 Da	8710.05 Da 8710.25 Da	12.17 min	7851.18 Da	7851.35 Da
siSOD1-36Sc	13.56 min 13.98 min	8694.86 Da	8694.86 Da 8694.20 Da	11.47 min	7851.18 Da	7850.92 Da
siSOD1-37	15.94 min 16.11 min	9004.91 Da	9004.75 Da 9004.89 Da	12.19 min	7851.18 Da	7850.92 Da
siSOD1-37Sc	14.38 min 14.49 min	8941.94 Da	8942.50 Da 8942.35 Da	11.51 min	7851.18 Da	7850.86 Da

**Table 3:** Physicochemical properties of siSOD1-peptide conjugates. (1) Retention time obtained with the following RP-HPLC conditions: buffer A: HFIP 12.5 mM and DIEA 4 mM in H_2_O and buffer B: HFIP 12.5 mM and DIEA 4 mM in MeOH. Flow rate was 0.3 mL/min with a gradient of 5–40% B in 18 min. The only exception was for the siSOD1-35 conjugate that was characterized in 12 min. (2) RP-HPLC-UV purity of both antisense (AS) and sense (SS) strands. (3) Identity is calculated as follows: |theoretical mass/experimental mass × 100| (4) Chip NiHC1000m; LDLR: 700–2000 RU; MCK mode; means ± SD of *n* = 6 determinations (triplicate analysis of 2 independent experiments).

	Code	siSOD1	Peptide Ligand	Characterization			Affinities
				Retention Time (min) ^1^	Purity (%) ^2^	Identity (%) ^3^	SPR (nM) ^4^
	VH4127	/	Pr-VH4127-NH_2_	/	/	/	40.1 ± 9.1
**5′P**	siSOD1-31	P-(5′AS)-siSOD1-(3′SS)-N6	Pr-K(N_3_)-PEG2-VH4127-NH_2_	Tr_AS_ = 12.64 min Tr_SS_ = 16.32–16.54 min	AS: 83.7% SS: 84.8%	AS: 99.99% SS: 99.98%	104.0 ± 11.7
	siSOD1-32	P-(5′AS)-siSOD1-(3′SS)-N6	Pr-VH4127-PEG2-K(N_3_)-NH_2_	Tr_AS_ = 12.24 min Tr_SS_ = 15.45–15.59 min	AS: 62.9% SS: 77.6%	AS: 99.98% SS: 99.99%	86.8 ± 17.1
	siSOD1-33	P-(5′AS)-siSOD1-(3′SS)-5-LC-NU	Pr-K(N_3_)-PEG2-VH4127-NH_2_	Tr_AS_ = 12.60 min Tr_SS_ = 16.15–16.40 min	AS: 89.5% SS: 85.1%	AS: 99.98% SS: 99.99%	12.8 ± 1.9
	siSOD1-34	P-(5′AS)-siSOD1-(3′SS)-N6	Pr-K(N_3_)-PEG2-VH4127-H_8_G_3_-NH_2_	Tr_AS_ = 12.64 min Tr_SS_ = 16.30–16.50 min	AS: 93.1% SS: 81.7%	AS: 99.98% SS: 99.99%	ND
**5′VP**	siSOD1-35	VP-(5′AS)-siSOD1-(3′SS)-N6	Pr-VH4127-G-OH	Tr_AS_ = 7.99 min Tr_SS_ = 9.30 min	AS: 88.2% SS: 96.3%	AS: 99.98% SS: 99.98%	11.3 ± 2.9
	siSOD1-36	VP-(5′AS)-siSOD1-(3′SS)-N6	Pr-K(N_3_)-PEG2-VH4127-NH_2_	Tr_AS_ = 12.17 min Tr_SS_ = 15.47–15.64 min	AS: 72.6% SS: 95.8%	AS: 99.98% SS: 99.99%	25 ± 1.2
	siSOD1-37	VP-(5′AS)-siSOD1-(3′SS)-5-LC-NU	Pr-K(N_3_)-PEG2-VH4127-NH_2_	Tr_AS_ = 13.19 min Tr_SS_ = 15.94–16.11 min	AS: 93.8% SS: 67.8%	AS: 99.98% SS: 99.98%	31.9 ± 1.6

**Table 4:** Summary of unconjugated siSOD1 and siSOD1-peptide conjugates in vitro and in vivo KD.

	Code	In Vitro KD on N2A		In Vivo KD
		Transfection	Free Uptake	Liver
**5′P**	5′P-siSOD1	>90%	20%	10%
	siSOD1-31	>90%	55%	40%
	siSOD1-32	40%	30%	15%
	siSOD1-33	>90%	45%	45%
	siSOD1-34	>90%	60%	15%
**5′VP**	5′VP-siSOD1	>90%	40%	10%
	siSOD1-35	>90%	40%	50%
	siSOD1-36	>90%	60%	30%
	siSOD1-37	>90%	60%	35%

## Data Availability

The data presented in this study are available in this article and Supplemental Materials.

## Supplementary Materials

The following supporting information can be downloaded at: https://www.mdpi.com/article/10.3390/pharmaceutics16040548/s1, Figure S1: Evaluation by SPR of VH4127 peptide and conjugate binding to LDLR. Figure S2: Validation of LDLR expression and VH4127 binding in the Neuro-2a cell line.
