# Supplementary material for: LDLR-Mediated Targeting and Productive Uptake of siRNA-Peptide Ligand Conjugates In Vitro and In Vivo
Source: Pharmaceutics. 2024 Apr 17;16(4):548. doi: 10.3390/pharmaceutics16040548 (PMC11054735; doi:10.3390/pharmaceutics16040548)
Supplement: Supplementary file 1 [file pharmaceutics-16-00548-s001.zip › Supplemental Figure S1 VF.pdf]

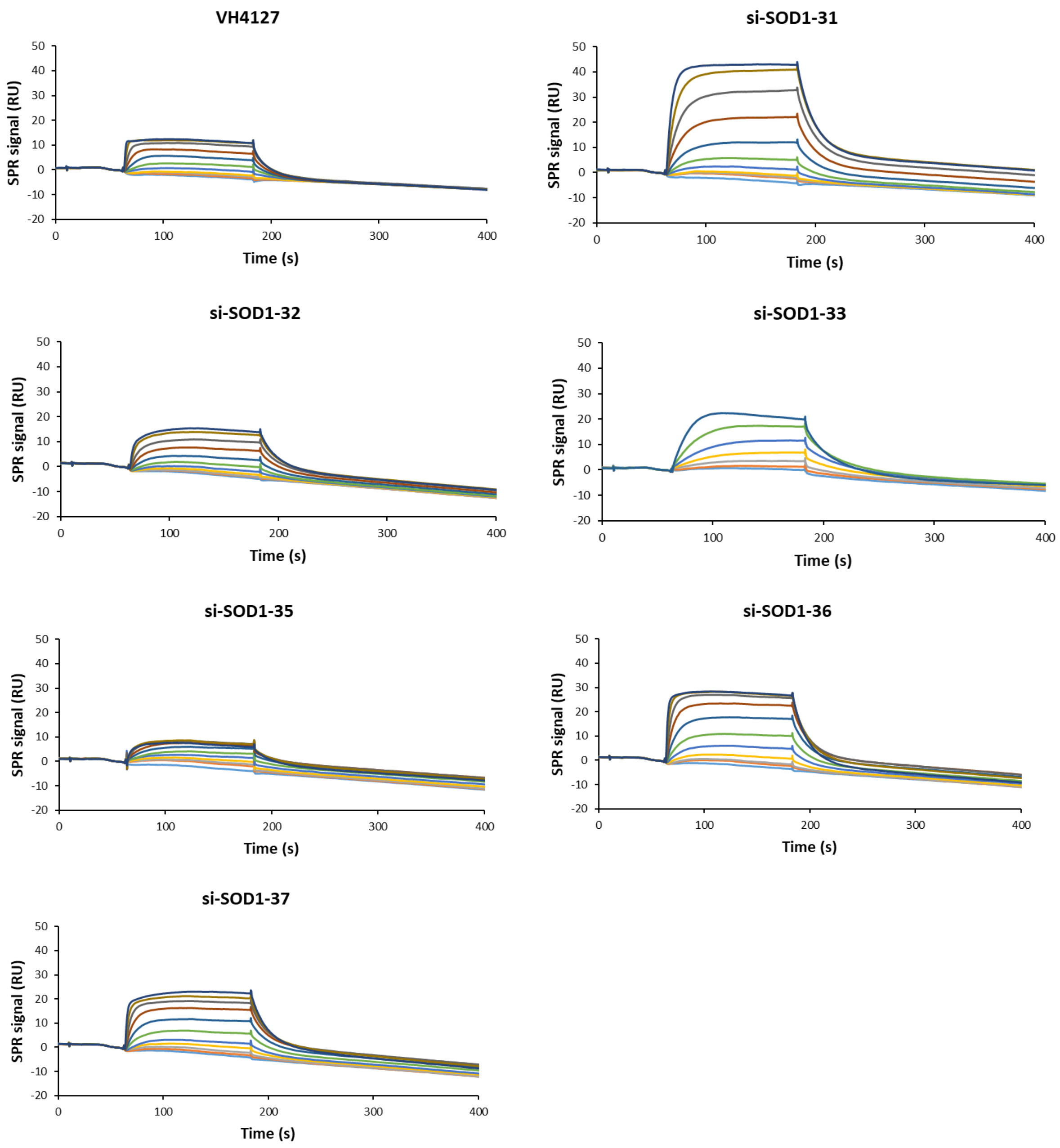

Supplemental Figure S1: Evaluation by SPR of VH4127 peptide and conjugate binding to LDLR. Increasing concentrations of VH4127 peptide or conjuguates (0.6-640 nM excepted for si-SOD1-33 0.6-40 nM) were injected over LDLR immobilized on Ni sensor chip. Typical sensorgrams are displayed. Each figure is representative of two independent experiments.
