# Supplementary figures and images for: LDLR-Mediated Targeting and Productive Uptake of siRNA-Peptide Ligand Conjugates In Vitro and In Vivo
Source: Pharmaceutics. 2024 Apr 17;16(4):548. doi: 10.3390/pharmaceutics16040548 (PMC11054735; doi:10.3390/pharmaceutics16040548)

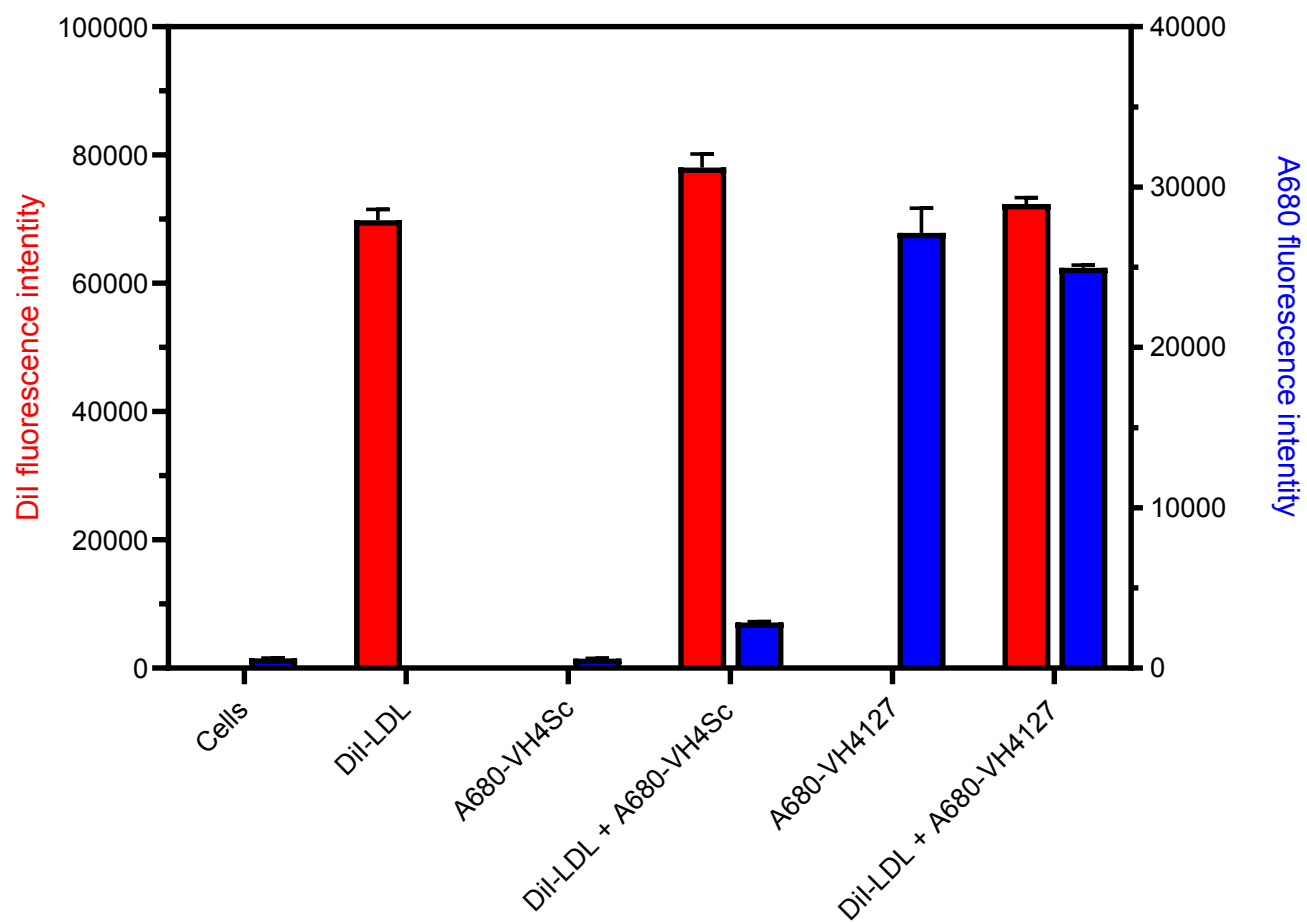

**Supplemental Figure S2:** Validation of LDLR-expression and VH4127 binding in the Neuro-2a cell line

Supplement: Supplementary file 1 [file pharmaceutics-16-00548-s001.zip › Supplemental Figure S2 VF.pdf]
